# Upper limits to sustainable organic wheat yields

**DOI:** 10.1038/s41598-021-91940-7

**Published:** 2021-06-16

**Authors:** Thomas F. Döring, Daniel Neuhoff

**Affiliations:** Agroecology and Organic Farming Group, University of Bonn, Auf dem Hügel 6, 53121 Bonn, Germany

**Keywords:** Agroecology, Plant sciences

## Abstract

Current use of mineral nitrogen (N) fertilizers is unsustainable because of its high fossil energy requirements and a considerable enrichment of the biosphere with reactive N. Biological nitrogen fixation (BNF) from leguminous crops is the most important renewable primary N source, especially in organic farming. However, it remains unclear to which degree BNF can sustainably replace mineral N, overcome the organic to conventional (O:C) yield gap and contribute to food security. Using an agronomic modelling approach, we show that in high-yielding areas farming systems exclusively based on BNF are unlikely to sustainably reach yield levels of mineral-N based systems. For a high reference wheat yield (7.5 t ha^−1^) and a realistic proportion of fodder legumes in the rotation (33%) even optimistic levels of BNF (282 kg N ha^−1^), resulted in an O:C ratio far below parity (0.62). Various constraints limit the agricultural use of BNF, such as arable land available for legumes and highly variable performance under on-farm conditions. Reducing the O:C yield gap through legumes will require BNF performance to be increased and N losses to be minimised, yet our results show that limits to the productivity of legume-based farming systems will still remain inevitable.

## Introduction

The question whether the global conversion of agriculture to Organic Farming (OF) systems would be able to supply the current and future demand for food and feed is the subject of scientific inquiry, public discussion, and political debate^1–3^. A rigorous analysis needs to distinguish between political, socio-economic and agronomic aspects of world food supply, before they are integrated into complex strategies. Although there is some agreement that the current food production level is principally sufficient to feed the approximately 7.8 10^9^ people living today, the number of people suffering undernourishment is unacceptably high^4,5^. However, the causes for food insecurity are to a large degree independent of farming systems and are mainly due to political and economic reasons^6,7^. In contrast, the production of calories and proteins needed for human nutrition as an agronomic issue mainly depends on the available agricultural area and its productivity, i.e. the yield level, but also on the stability of a cropping system. Any scenario on effects of global conversion to OF on food production needs to be based on a realistic estimation of attainable yields. In this context, the productivity of cereals is of primary importance due to their dominant role for world food supply^8^.

**Figure 1:**
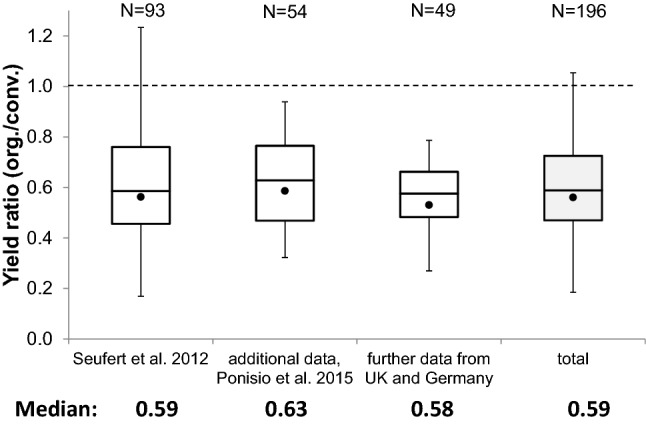
The wheat yield gap. Ratio of organic to conventional wheat yields (O:C ratio) from various data sources. Quartiles (box), mean (filled circle), 2%- and 98%-percentile (whiskers); absolute maximum (1.76) and minimum (0.13) not shown; number of observation pairs given at top. The second box shows data compiled by Ponisio et al.^11^ that was not included in the meta-analysis by Seufert et al.^10^. Further data not included in either study is shown in the third box.

**Figure 2:**
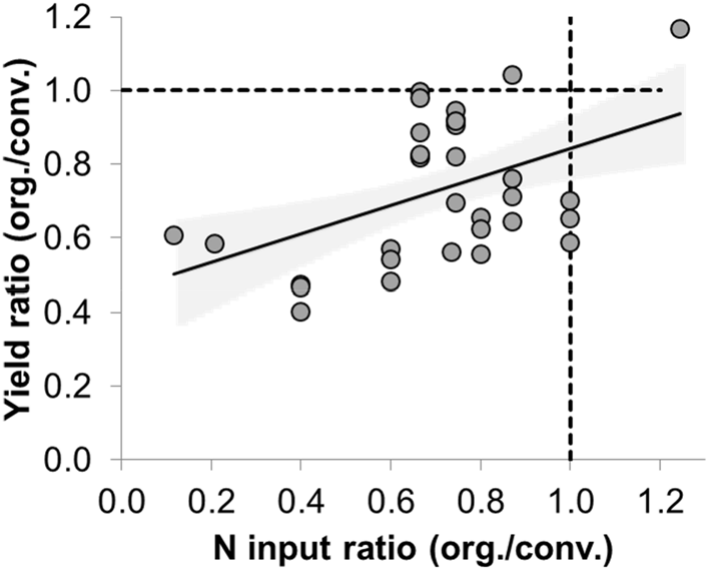
Dependence of the O:C yield ratio on nitrogen input. Based on data compilation by Ponisio et al.^11^; cases restricted to those where N input was 120–150 kg N ha^−1^ in conventional system.

A recent meta-analysis compared conventional and organic cereal yields of 156 data sets. Cereal yields in organic systems were on average 78% of those obtained in conventional systems^9^. Yield differences showed a wide range, e.g. 40–130% for wheat (average = 73%). Two further meta-analysis confirmed these estimations (Fig. 1). Across 161 comparisons, organic cereal yields were 26% lower (40% for wheat) than conventional ones^10^. In another meta-analysis (n = 559) organic cereal yields were 22% lower than under conventional management (37% for wheat)^11^. O:C ratios generally get closer to parity with increasing nitrogen input in organic systems (Fig. 2), i.e. when the N-input ratio in both systems is close to 1. From a methodological point of view, however, the O:C ratios cited here are questionable and therefore at least partly inconclusive.

The two main problems related to the validity of yield comparisons are the determination of an adequate reference system, and the proper selection of representative production techniques. Paired comparisons of individual crop yields are generally only of limited validity if yield only refers to single crops and ignores the crop rotation^12^. Organic cereals grown after a two-year legume ley with additional farmyard manure (FYM) application, for example, may have a similar N supply as a conventional crop^13^. Subsequent organic crops in contrast will have a lower N-supply resulting in decreasing yield levels over the rotation. While organic farmers may then choose less N-demanding crop species later in the rotation, a direct comparison with a much simpler conventional rotation is difficult. Many studies comparing yields from organic and conventional systems, however, have ignored rotational constraints, in particular, in terms of N supply from legumes to cereals.

The validity of a study is also limited if the empirical data has been generated using non-representative amounts of nutrient inputs. Unfortunately, the approach of applying high amounts of FYM or other organic inputs to organic crops not generated on the farm (substitution method) is commonly practiced in research^14,15^, and this hampers the generalisation of yield comparisons, as organic practice is much more limited in N supply. There is also some evidence that O:C yield gaps calculated on the basis of field experiments are lower than those found under on-farm conditions^16^.

For meaningful comparisons, it is also important to distinguish between actual and attainable yields. Comparisons of actual yields, which are determined by biotic stress and limited nutrient and water supply in farmer fields, may sometimes result in low or no organic to conventional yield gaps. They are mainly a function of the specific degree of suboptimal management in the systems under comparison. Under conditions of extensive non-organic agriculture with missing access to agricultural inputs, conversion to OF may therefore lead to equal or even higher actual yields compared with conventional production^3^. From an agronomic perspective, however, comparisons also need to include the attainable yield, where water and or nutrients are non-limiting and biotic stress is controlled^17^. Because of these various shortcomings, a realistic estimate of the potential of organic and other legume-supported cropping systems is currently lacking. We therefore aimed to include aspects of rotation and N balance into an assessment of legume-supported crop productivity.

Here we propose a novel deductive method for calculating an *upper limit* of sustainably achievable O:C ratios for wheat for a typical cropping system in a temperate climate, assuming that nitrogen is the decisive yield limiting factor in organic cereal production. To be sustainable, rotational nitrogen budgets in OF must be balanced, i.e. nitrogen supply from within the system needs to match nitrogen demand of all crops.

Thus, our aim is to determine an upper limit of wheat yields that can be maintained sustainably by legume-derived nitrogen. By this it is possible to quantify O:C ratios for a crop species paradigmatic for world food supply and hence suitable for world nutrition scenarios. Because we aim at an upper yield limit we follow the basic principle to assume optimal conditions for the legume-supported organic system. The complex dynamics of nitrogen flows through agroecosystems are therefore simplified in the model. An important component of our approach was to choose some key conditions to ensure that the yield of the legume-supported cereal in the model will always be greater than an actual realistic value. The spatial level of our scenarios is an aggregate of individual farms at the regional level. By taking the availability of arable land into account, other than with many previously published pairwise O:C comparisons, the essential aspect of land use competition is included in our model.

## Results

The O:C ratio is defined as the quotient of the calculated total cereal production (either dry matter = dm or energy = en or protein = pr) of a given organic arable land area relative to the total conventional production that would be harvested from the same area. In our model, upper limits of organic cereal yields are determined by the N-supply generated by BNF during the crop rotation. We assume that the total BNF will be derived from fodder legumes since on average these have higher BNF rates than grain legumes. Nitrogen fixed by the legumes is assumed to be fully available for the non-legumes without losses (see below), defining a theoretical ceiling. Wheat is used as an example to represent non-legume crops, though it needs to be considered that other crops such as rye or oats will show differing responses to nitrogen (see Supplementary Material S1, Tables S3 and S4).

**Figure 3:**
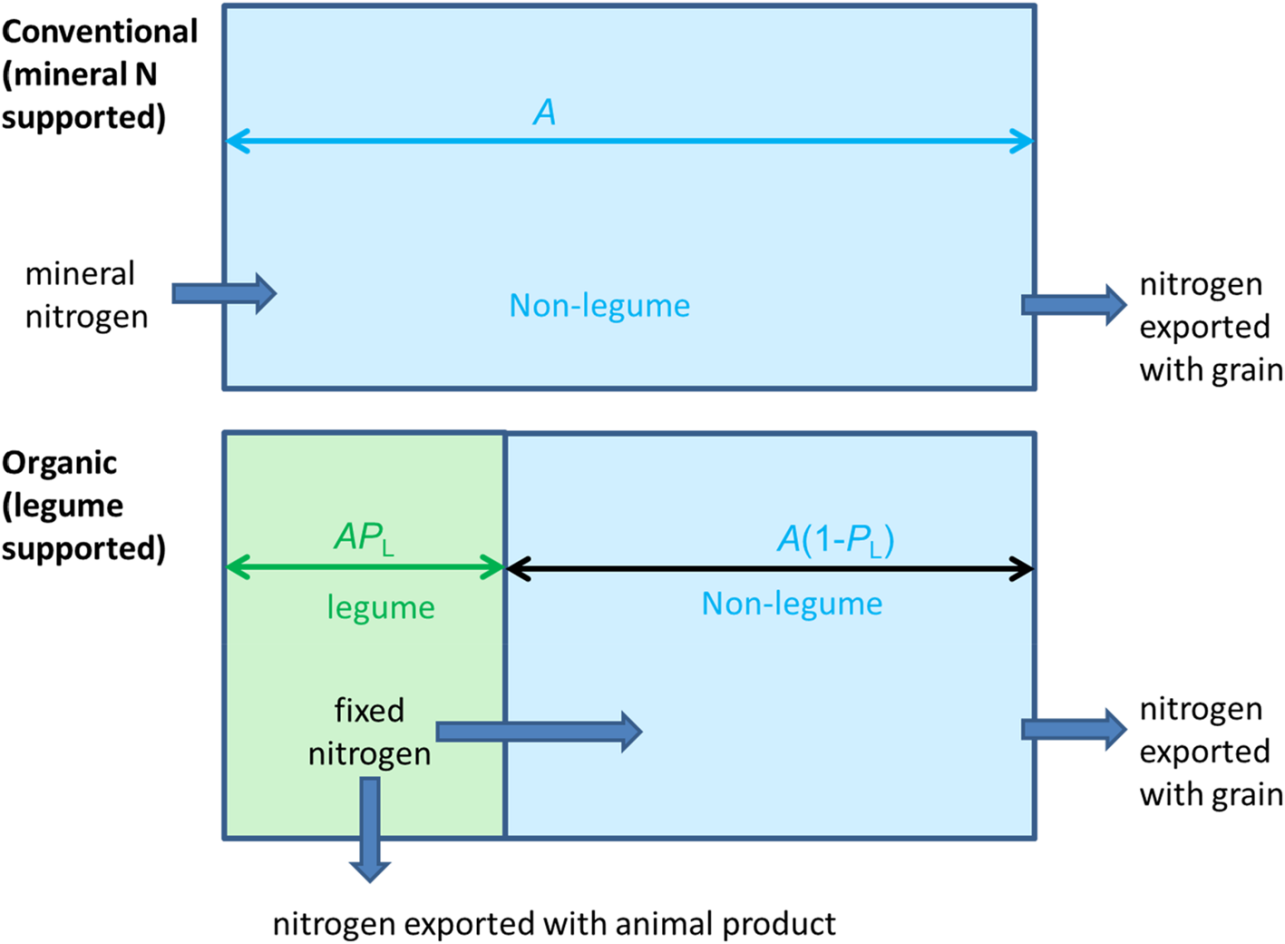
Simplified representation of the model.

One of the main model input variables is the proportion of legumes in the rotation. As this proportion increases, more nitrogen becomes available for the non-legume, while the land area on which the non-legume can be grown is reduced (Fig. 3). The model shows the net effect of these two mechanisms with different scenarios.

**Figure 4:**
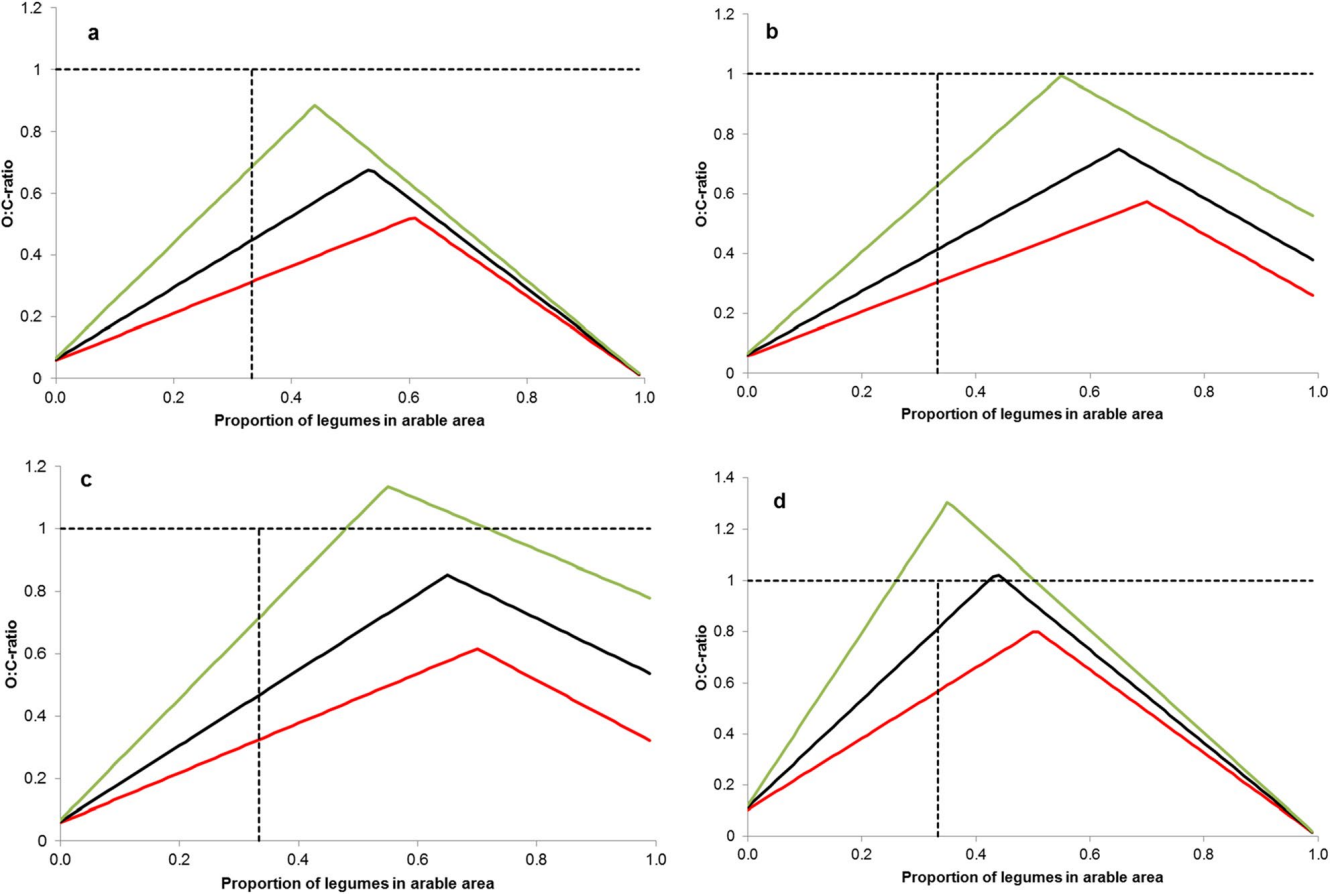
Modeled maximum O:C ratio depending on proportion of legume in arable area. Optimistic scenario (green lines), realistic scenario (black lines), pessimistic scenario (red lines). Dashed vertical line indicates recommended proportion of forage legumes in rotation. O:C ratio calculated for cereal yield only (**a**), total energy output from wheat and forage legume via conversion to milk (**b**,**d**); and for total protein yield (**c**); references level of high (**a**–**c**) and lower (**d**) conventional wheat yield levels.

Major factors determining O:C are the conventional reference yield level, the proportion of legumes (P_L_) in the rotation and the BNF performance of the legumes (Fig. 4); for further analyses on effect of grain N content, non-BNF balance of nitrogen, and N saturation point on O:C see supplementary material, Fig. S1–S3. When assuming a high legume BNF (282 kg N ha^−1^ a^−1^) and a reference conventional cereal yield level of 7.53 t ha^−1^ (86% dm) a maximum O:C_dm_ of 0.89 will be obtained with a legume proportion of P_L_ = 48% of the arable land area (Fig. 4a). Maximum O:C_dm_ for lower values for BNF (183 or 132 kg N ha^−1^ a^−1^) require higher P_L_ values (55 to 60%) and are even lower (O:C_dm_ between 0.52 and 0.68) than with high BNF. When assuming a more realistic P_L_ in an organic rotation of 33% and still a high BNF, the upper limit for the O:C_dm_ becomes lower (0.68). In farming practice, capping the proportion of legumes is an agronomic necessity because of the low self-compatibility of legumes (see “Discussion” section).

The corresponding O:C_dm_-values for medium and low BNF for P_L_ = 33% are still lower and range between 0.31 and 0.44. As P_L_ increases beyond the optimum, O:C_dm_ decreases are due to the nitrogen saturation point, where further nitrogen supply will not produce higher cereal yields.

Because the calculation considers only the cereal O:C_dm_ of a given area, and fodder legume biomass is not included, increasing values for P_L_ will lead to a lower cereal production, up from the N-saturation point, because while cereal yield remains constant from that point onwards, cereal area decreases with increasing P_L_. Accordingly, the values presented up to here just allow a statement on the O:C_dm_ of cereals in fodder legume based versus pure cereal based crop rotations.

However, comparing O:C based on the dry matter production of cereals only does not account for the caloric value of the fodder legumes, e.g. when converted to milk via ruminants. When including the caloric value of cow’s milk in the model, the resulting O:C ratio refers to overall energy output (O:C_en_). Compared with O:C_dm_, a higher O:C_en_ can be obtained for the same conventional reference yield level of 7.53 t ha^−1^ (Fig. 4b). The O:C_en_ equals 1 if BNF is high (282 kg N ha^−1^ a^−1^) and P_L_ = 55%. Maximum O:C_en_ values for medium and low BNF require proportions of legumes above 60%. Interestingly, the O:C_en_ values at 33% P_L_ are lower than the corresponding values for O:C_dm_ (Fig. 4a vs. b). This is due to the fact that nitrogen leaving the system via milk is no more available for the cereals, while for O:C_dm_ all N derived from BNF was considered available for the cereals. When assuming only one third of the arable land to be grown with fodder legumes, O:C_en_ becomes substantially lower and ranges between 0.62 (high BNF) and 0.23 (low BNF).

Limiting the O:C to energy produced by the two systems only might not be an appropriate comparison, since the high quality milk proteins are not considered. However, a comparison of O:C_pr_ (protein yield ratio) shows the same trend as O:C_en_, but on a slightly higher level (Fig. 4c). The maximum O:C_pr_ is > 0.7 (at P_L_ = 33% and the optimistic scenario) underlining the comparatively high protein value of milk. Calculating O:C_pr_ with medium or low BNF results in an over-proportional decrease of O:C_pr_ but values are still higher than for O:C_en._

All O:C ratios presented above were calculated based on a comparatively high conventional reference yield level (7.53 t ha^−1^). This corresponds to the 95%-percentile of global wheat yields (FAO 2015), but simultaneously is also the 20-year average of wheat yields in Germany. When assuming a conventional reference yield level of 4.2 t ha^−1^ only, i.e. the 75% percentile of global wheat yields, O:C_dm_ becomes substantially higher independent of the level of BNF. For high BNF, an O:C_dm_ > 1 would already be obtained with PL < 0.33. Medium BNF at a PL of 33% would still result in an O:C_dm_ of > 0.8 versus 0.56 for low BNF (Fig. 4d). This is also confirmed when wheat is replaced with oats, which has a much lower reference yield level than wheat (see Supplementary Material, Table S4). However, this scenario (low reference yield level with high OC-ratios) comes at the cost of relatively low absolute yields of the legume supported cereal.

**Figure 5:**
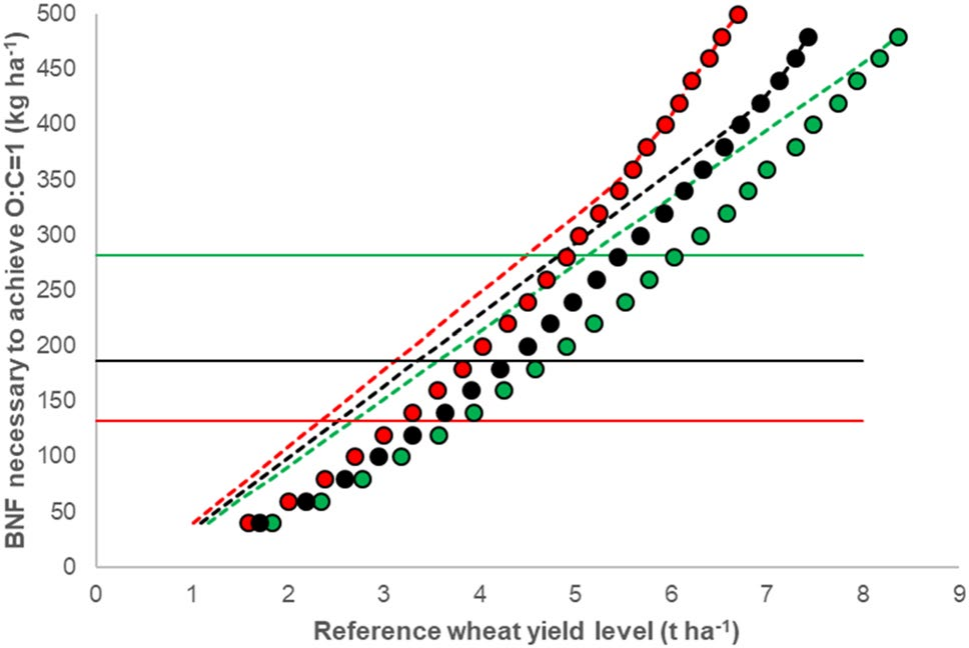
Biological nitrogen fixation (kg ha^−1^ N) necessary to achieve a yield OC-ratio of 1 depending on conventional reference yield level. Proportion of area grown with legume flexible (symbols) or capped at 0.33 (dashed lines); removal of N with the wheat grain from the field (as determined by the percentage of N in the grain) is low (green symbols and dashed lines), medium (black) or high (red). Horizontal lines correspond to upper quartile (green), median (black) and lower quartile (red) of empirical BNF data.

The general relationship between the three key factors affecting O:C, namely BNF, conventional reference level and proportion of legumes in the land, is shown in Fig. 5. When limiting the proportion of fodder legumes to a proportion of 33% of the land, the maximum O:C_dm_ shows a close correlation with BNF and the cereal reference yield (Fig. 5). Assuming a moderate N content in the grain, already a reference yield level of 5.0 t ha^−1^ would require a BNF of 293 kg ha^−1^ for achieving O:C_dm_ = 1. At low reference yield levels of < 3.0 t ha^−1^ an O:C_dm_ of 1 could already be obtained with a P_L_ of 33% and a BNF of 163 kg ha^−1^.

## Discussion

To our knowledge, in the literature on organic to conventional (O:C) yield gaps, approaches assessing the yield potential based on nitrogen balances have not been published so far. The approach for calculating O:C ratios presented here is based on the sustainability concept of OF and the assumption that the productivity of cereals in most organic cropping systems is limited by nitrogen availability^13,18,19^. To ensure short-term productivity and long-term sustainability of OF it is important to maintain soil fertility by achieving a balance between nutrient input and output. Field level nutrient budgets, which consider the differences between total inputs and removals from a field are used to determine nutrient requirements of a single crop. For sustainable yields, however, nutrient management and budgeting need to be understood and planned over rotation cycles^20^. System budgets—with data on nutrient losses and internal flows within a rotation cycle—are more suitable to evaluate the sustainability and productivity of a cropping system. Sustainable yields can only be attained if the nutrient budget, in particular for N, of the system is balanced^21^.

Long-term yields of organic non-legume crops are determined by the net nitrogen input across the crop rotation. The only relevant net gain of nitrogen for organic cropping systems is symbiotic BNF provided by legumes. Leaving legume cultivation out of organic crop rotations inevitably leads to soil N-depletion and in the medium-term to decreasing yields. Other nitrogen inputs such as deposition and non-symbiotic BNF only play a minor role and were estimated for our model with 20 and 5 kg N ha^−1^ a^−1^ respectively, although some authors consider the latter to be higher^8^. Non-legume crop residues as well as FYM application delineate the temporary field balance’s nutrient turnover, but not the long-term system N-balance. External nitrogen sources, such as a purchased manure only play a role for individual farm nutrient budgets^21^, but are not relevant on a larger scale. In a simplified model, organic yields are therefore determined by the productivity of legume based cropping systems.

The performance of BNF is mainly a function of the proportion of legumes in the rotation, legume total dry matter production and the percentage of nitrogen derived from the atmosphere^22^. Net contribution to the soil nitrogen balance further depends on legume use either as green manure, fodder for livestock or cash crop. The net soil nitrogen input of grain legumes used as cash crops is generally low unless the nitrogen harvest index is low^23^. To delineate the upper boundary of O:C it is therefore reasonable to refer to forage legumes known to have the highest BNF performance of legumes^24^.

Our model compared a high and medium conventional reference cereal yield level with three scenarios of BNF productivity (282, 183 and 132 kg N ha^−1^ a^−1^) based on forage legumes. For the assumption of high reference yield levels (7.53 t ha^−1^ 86% dm) we demonstrated that even with high BNF and a P_L_ of 33% the upper boundary of O:C_en_ is 0.62. Higher O:C ratios can only be achieved under conditions that do not seem feasible on a large scale. A recently published yield comparison between conventional and organic systems in the Kenyan highlands, for example, showed comparable corn yields for high input systems^14^. Grain yield of sole cropped organic maize with an N-input of 96 kg ha^−1^ was on average of two cycles 5.14 t ha^−1^ versus 4.89 t ha^−1^ (n.s.) under conventional management. The total annual N input over two seasons and crops (corn and cabbage) amounted 251 kg N ha^−1^. Organic inputs were based on composted farm yard manure and both *Tithonia* mulch and tea taken from hedges and wild collection. Such an approach is feasible for individual organic farms but might reach a limit on larger scale due to resource unavailability.

In this context it is interesting to compare the model-derived yield ratio (O:C_dm_) (Fig. 4d), with the empirically derived O:C-ratio for wheat yields in the meta-analyses (Fig. 1). When the proportion of legumes was restricted to practically sustainable levels of P_L_ = 0.33, the model showed, under realistic and favourable conditions maximal O:C-ratios of 0.44 and 0.68, respectively. We can also calculate corresponding ‘unprotected’ O:C-ratios that do not consider the availability of land for legumes vs. cereals. These values are 1.22 and 1.52, respectively, and are, as expected, much higher than the overall median of empirical yield O:C-ratios, of 0.59. While many factors may contribute to discrepancies between the different approaches, it is likely that organic wheat in the empirical studies experienced additional yield losses via pests, weeds and diseases which were not included in our model. Simultaneously, it is likely, and confirmed by Fig. 2, that N inputs in the organic relative to the conventional systems will have played a major role in determining the OC-ratio in the empirical studies.

Our results show that the O:C ratio strongly depends of the conventional reference yield level. Lower reference yields mathematically result in narrowing the O:C ratio. However, independent of the cause for low conventional yield levels, the attainable yield O:C gap may remain relatively wide. If the actual conventional reference yield is limited by nitrogen, higher conventional yields could be attained just by N-fertilizer application. If site specific factors, e.g. soil or climatic conditions, especially water availability limit cereal yield, it is likely that correspondingly, legume production and BNF will be low as well, compensating the effect of low conventional reference yields and thereby resulting in relatively low O:C values. Optimistic estimations about the potential of legume supported cropping systems to feed the world^2^ assuming maximum O:C ratios of 0.75 appear somewhat doubtful against this background.

The O:C ratios suggested here refer to wheat, one of the three cereals predominant for world nutrition. Theoretically, higher maximum O:C ratios could be attained for crop types with a lower N demand (also see supplementary material), and this is supported by yield comparisons^25^; for rice, which is less N demanding than wheat, using green manure crops or *Azolla* water fern is known to have a potentially considerable nitrogen fertilizer equivalent^26,27^. These approaches have been neglected during the past decades because of the large scale adoption of mineral nitrogen fertilizers. *Azolla* water fern, for example is estimated to only play a minor role in global paddy rice production^24^. The basic interdependence of those systems with the requirement of balanced N-budgets and the permanent competition for arable land use however remain unaltered, also for crop categories other than cereals such as oil seeds and vegetables.

The O:C_en_ ratio of 0.62 calculated for high BNF of 282 kg N ha^−1^ a^−1^, 33% P_L_ and a high reference wheat yield level typical for Western Europe delineates the nitrogen limited theoretical maximum value assuming no losses during the internal nutrient flow.

Under on-farm conditions, however, the amount of BNF-N available for non-legumes will often be lower than this level for two main reasons. First, organic forages are generally mixed swards of legumes and grasses for reasons of yield stability and fodder quality which may, depending on the legume proportion in the mixture, result in a reduction of BNF-N input into the soils^22^. Second, and more importantly, legume-based cropping systems are linked with unavoidable N-losses from the system. Recycling of legume nitrogen harvested on the field via animal feeding, subsequent manure production in the stable and final broadcasting on the fields results in N-losses which can range between 17 to 46 kg N cow yr^−1^ ^28^. Further nitrogen losses relevant for the system budget may also occur from leaching ^29,30^.

Moreover, the actual performance of BNF is often restricted by abiotic factors such as temperature, water and nutrient supply^31^, in particular phosphorous^32,33^, sulphur^34^ and molybdenum^35^. Biotic constraints such as abundance and persistence of specific *Rhizobium* strains in the soil and soil borne diseases such as *Sclerotinia trifoliorum* may additionally limit rotational BNF performance^36^. While many of these issues may be solved through fertilization, irrigation or microbial inoculation, it is likely that even under ideal legume management, BNF will often remain below the high level assumed in our optimistic scenario.

Organic cereal yields not only depend of the rotational BNF input. The actual yield in each field further depends of the temporal N-availability over the season. The primary determinants for N-availability are the net rate of N release from SOM, the contributions from organic and inorganic N-sources such as FYM and losses from the plant available N pool^37^. Favourable environmental conditions and adequate management may easily result in high nitrogen supply and a positive N-balance of an organic field, when combining pre-crop effects of ley with manure application. However, unfavourable environmental conditions such as drought and cold often result in low soil nitrogen mineralization and subsequent lower productivity of organic cereals. Due to the trend of delayed N release from legume residues, cereal N demand in the early season is often not adequately matched^38^. The missing synchrony between crop demand and N supply from the soil tends to result in a lower crop N recovery of organic fertilizers^18^ and partly explains the high variation of actual yields in OF.

For the ultimately attainable O:C ratio biotic factors reducing cereal yields need to be considered as well. Global estimates for wheat carried out for 19 world regions over a period of three years 2001 to 2003 showed considerable actual yield losses of 28.2%^39^. Pathogens (10.2%) were the main cause for actual yield losses in wheat. The few comparative studies on disease incidence suggest that there is a lower disease pressure in cereals in low-input compared with intensive conventional systems with fungicide use^40^. Site-specific climatic conditions however may also lead to fungal epidemics in organic cereals, e.g. rust diseases, if resistance levels are insufficient, as no curative fungicides are available.

In contrast, several effective control options are available for organic pest and weed management. In addition to preventive measures, pests in organic crops can often be controlled by using specific approved pesticides^41^. Weeds, often considered to be a major challenge in organic crop production can be managed with a wide range of indirect and direct control methods, though some are not always economically viable^42^.

Apart from the single farm-oriented view on O:C as discussed above, the global dimension of a full conversion scenario needs to be considered as well. Since the end of the Second World War the global use of mineral nitrogen fertilizers has steadily increased. Together with animal manure they constitute the major input of nitrogen in conventional arable land^43^. The total global nitrogen input in agricultural soils managed as both cropland and grassland was estimated to be ~ 249 Tg in the year 2000. At that time, mineral nitrogen already covered one third of the input ~ 83 Tg, and in 2013 already 107 Tg^44^, while the contribution of BNF was only ~ 30 Tg^43^. Only 93 Tg were removed with the harvested crops resulting in a considerable enrichment of the biosphere with reactive N.

When assuming that (i) the major part of mineral N fertilizer is applied to arable land, (ii) the current production level of staple crops needs to be at least maintained and (iii) the N use efficiency (43%) remains unaltered, (iv) the average global BNF amounts to 165 kg N ha a^−1^ ^24^, the replacement of ~ 83 Tg N would at least require some ~ 500∙10^6^ ha of fodder legumes, which corresponds to approximately one third of the current total global arable land area of ~ 1.5 10^9^ ha.

In countries with a high population density and low availability of arable land, e.g. Bangladesh or Indonesia, this scenario currently seems improbable. Intensive, often unsustainable rice production systems for example in Indonesia with up to three seasons per year produce high amounts of caloric energy in a short period and cannot be based on legumes only, unless accepting a significant reduction of the production, or a global shift in human diets. For this reason mineral N has been considered to be indispensable for middle and low income countries such as India, China and Indonesia, which have achieved staple food self-sufficiency^45^. Some authors claim that human population now exceeds the carrying capacity of agricultural systems that exclusively depend on legumes for N input and that a sizable percentage of the world population depends on mineral N-fertilizers^46^. At the same time, however, caution is due when applying the results from our study across the globe. The data feeding our models was mostly collected from temperate climates and transferability to tropical regions is limited, not only for ecological and agronomic but also for socio-economic reasons.

Exclusively legume based cropping systems require time and space for growing fertility building crops and may therefore be best suited for areas where cropland availability per capita is high. Growing more legumes has been repeatedly claimed for crop management schemes that aim at enhancing sustainability and buffering against the dependence of mineral N-fertilizer^46–48^. The current political will to significantly reduce greenhouse gas emissions is expected to feed a greater interest to reduce mineral N-fertilizer input by using legumes. Experiments in the USA have shown that including alfalfa in a cereal rotation can help to reduce the N-fertilizer need by 25%^49^. In the tropics BNF can significantly contribute to reduce dependence of mineral N fertilizers, if there is no competition for arable land^50^ and soil P-supply not limiting, which, however, is not the case for some parts of the world^51^.

## Conclusions

In many cases, sustainable OF systems will have lower attainable yields than current conventional systems, even with good management practices. According to our modelling, average O:C values for cereals cited in the literature are likely to overestimate the sustainably maintainable productivity of OF. In return, OF produces a higher process quality for environmental impact categories such as biodiversity^52–54^, animal welfare^55^ and water nitrate contamination^56^ especially in regions with intensive farming. Permanent and intensive use of key inputs such as mineral nitrogen fertilizers and herbicides in simplified cropping systems is neither sustainable nor ecologically sound^7,57^. Consequently, a more ecological management of agroecosystems is globally required^58,59^. Many routine practices in OF could help to increase the resource efficiency and resilience of conventional production systems, including crop diversification using legumes and regular application of farmyard manure.

More research is needed to increase the productivity of OF systems and the adoption of BNF. Promising approaches include plant breeding, improved management of biotic and abiotic constraints, reducing N-losses from the systems and recycling nutrients from human waste. However, squeezed between the general limitations to sustainably maintainable organic yields on the one hand, and the environmental unsustainability of mineral N input on the other, approaches to meet the current and future demand for calories and protein are likely to depend on adjusting consuming behaviour with respect to dietary patterns^5,60^ and food waste as well as reduction of nutrient losses, especially through higher nitrogen use efficiency and improved nutrient recycling.

## Material and methods

### General model set up

The total production *Y*_Mo_ of cereal in the legume-supported system is the product of the yield *y*_Mo_ of the cereal (amount of grain per unit area) and the area available for the cereal. We divide a given area *A* of arable land into a defined proportion (P) grown with a legume (L) crop (*AP*_L_), while the remaining area *A*(1 − *P*_L_) is available for the cereal, so that *Y*_Mo_ = *y*_Mo_ (*1* – *AP*_L_) (Fig. 3). We then use published data to estimate the amount of nitrogen *n*_L_ that is generated on the legume area. This amount of nitrogen is the sum of N provided through BNF by the legume (*b*), and other sources, namely non-symbiotic nitrogen fixation (*d*), and atmospheric N deposition (*f*), minus losses (*l*): *n*_L_ = *b* + *d* + *f* − *l*. The N from legumes that is generally available for the area of the non-legume is then *m* = *n*_L_ *P*_L_/(1 − *P*_L_). This equation expresses the central notion of our model that with decreasing legume area, the amount of N from the legume area will not only be lower because BNF is derived from a smaller area, but also because it is then spread over a larger non-legume area.

We assume N to be the main yield limiting factor for the non-legume crop, and the entire N from the legume to be available for the cereal (see explanations below). We then calculate the legume-supported yield of the cereal, based on data of N content *u* in the cereal grain. In effect, as the grain N content decreases, the same amount of N can support an increasing grain yield level. Further, there is an N saturation point, i.e. an amount of N at which a further increase in available N does not lead to any further increase in yield.

Consequently, the upper limit of the cereal yield in the legume-supported system is *y*_Mo_ = *y*_Mz_ + *m*/*u*, if *m* < *s* and *y*_Mo_ = *y*_Mz_ + *s*/*u*, else, with *y*_Mz_ being the yield at zero N input, and *s* being the saturation point. The yield-O:C-Ratio *R*_Y_ for cereals is then the ratio of the production in the legume-supported system (*Y*_Mo_) to the production in the conventional system (*Y*_Mc_), i.e. *R*_YM_ = *Y*_Mo_ / *Y*_Mc_. Note that this ratio is not a ratio of yields but takes the available land for the cereal into account as well. The full model is given in the Supplementary Material (S1, model 1).

Because forage legumes such as clover can be converted to food such as milk and meat, we further calculate a second O:C ratio *R*_E_ where contributions from the legume and the cereal are expressed as energy and summed up for the cereal and the legume part of the rotation. This model is provided in the Supplementary Material (S1) as model 2.

### Main assumptions of the model

The main assumptions of the model are summarized below; further assumptions are detailed in the Supplementary Material (S1).

1. The **entire N from biological N fixation (BNF) is available** for the non-legume crop in the rotation. This represents the upper limit of the contribution of BNF to the non-legume yield. Typically, only a part of the N derived from BNF is available for the non-legume crop, e.g. because of nitrate leaching or gaseous losses of N. Note that for forage legumes, where part of the N derived from BNF is first exported from the field with the harvested or grazed plant biomass, we assume that this exported N is returned to the non-legume crop in its entirety through animal manure, but minus the fraction that is removed from the system via sold animal products, i.e. milk.
2. Because we impose the restriction of long-term sustainability, **N from turnover of soil organic matter (SOM) does not enter** as a net N source for the non-legume crop. This is to satisfy the condition that in the long term, the agricultural management needs to keep the SOM levels constant or increasing, not only because SOM is a source of N for the crop and may not be depleted, but also because of its other multiple benefits for soil fertility and crop growth. Following the same consideration, the model assumes that **N in straw** or in other products not entering human food chain completely remains in the production system, i.e. there is no net loss of N from non-legume plant residues. Straw either remains on the field, is incorporated into the soil through tillage, or is returned to the soil after being used in the stable. This assumes there is no loss of N from straw during or after usage in the stable. This overestimates the amount of N available from the crop via recycling of (non-legume) residues, again following our aim to determine upper sustainable legume-supported yield levels.
3. N **exported by harvested part of the non-legume crop (e.g. wheat grain) is lost completely** from the system. This assumption reflects the current situation that in most cases nutrients from grain of wheat and other cereals, when used as food, are not returned to the field through nutrient recycling of food waste and human waste back to the field. In our scenarios, this fraction of N ultimately exported with the non-legume harvest is to be covered by BNF.
4. The **conventional** reference production system includes **no legumes**. Although legume cropping is currently being taken up to an increasing degree by non-organic farmers, proportions are still relatively low (< 10%) and this is mainly restricted to grain legumes.
5. Unavoidable **N-losses** within the system through volatilization and leaching are included under the assumption of **minimal** losses.

### Data sources

Main input variables of the model are listed in Table 1. Further values in the model were fixed and included atmospheric nitrogen deposition (*d*) at 20 kg ha^−1^ a^−1^ N^61,62^; non-symbiotic N fixation from free-living bacteria in the soil (*f*) at 5 kg ha^−1^ a^−1^ N ^24^; unavoidable losses of N from the soil (*l*) at 15 kg ha^−1^ a^−1^ N; and N content of organic milk at 0.57%. Data with all sources are compiled in Supplementary Material (Data file S2).

**Table 1:** Input data used for modelling.

Variable	Explanation	Unit	Percentiles				Number of values
			25%	50%	75%	95%	
*b*	Biol. nitrogen fixation	kg N ha^−1^ a^−1^	132	186	282	430	218
*u*	N content of wheat grain dry matter	%	2.2	2.1	2.0	1.7	39
*y*_Mc_	Conventional yield	t ha^−1^	2.1	2.9	4.2	7.5	83
*s*	Saturation point	kg N ha^−1^ a^−1^	149	190	258	334	58
*y*_F_	Forage yield dry matter	t ha^−1^	6.7	10.0	12.5	17.8	158
1/z_µ_	Forage needed for milk	kg kg^−1^	1.35	1.38	1.48	(1.53)	9
*x*_µ_	Energy content of milk	MJ kg^−1^	2.71	2.76	2.92	3.19	10
*x*_M_	Energy content of wheat	MJ kg^−1^	13.0	13.7	13.9	14.5	33

### Optimistic, realistic and pessimistic scenarios

Our approach to deal with variability in input data is to use deterministic models under three scenarios. The ‘optimistic’ scenario assumes that *all* input variables are simultaneously in favour of the legume-supported (organic) system, and uses the upper quartiles (75%-percentile) of all variables. The ‘realistic’ scenario uses all the medians (50%-percentile) from the input variables, whereas the ‘pessimistic’ scenario takes the lower quartiles (25%-percentile). Thus, the optimistic scenario provides an estimate of what O:C ratio can be achieved if conditions (e.g. for BNF) are generally advantageous; this approach ignores potential trade-offs, i.e. when one input variable is unlikely to be high if another input variable is also high. Not taking such trade-offs into account follows our aim to determine an *upper limit* of yields.

## Supplementary Information

Supplementary Information 1.

Supplementary Information 2.
