# Supplementary material for: Upper limits to sustainable organic wheat yields
Source: Sci Rep. 2021 Jun 16;11:12729. doi: 10.1038/s41598-021-91940-7 (PMC8209060; doi:10.1038/s41598-021-91940-7)

# Supplementary information for

Upper limits to sustainable organic wheat yields

**Thomas F. Döring & Daniel Neuhoff**

# Assumptions and conditions of the model

Table S1: Abbreviations

| **Index or abbreviation** | **Meaning** |
| --- | --- |
| L | Legume |
| M | Non-legume (‘main crop’), e.g. cereals |
| O | Organic |
| C | Conventional |
| E | energy |

## Model 1. Organic : conventional ratios for non-legume yields

Table S2: Variable explanations for Model 1

| **Variable explanation** | **Unit** | **Symbol** |
| --- | --- | --- |
| concentration of N in cereal grain dry matter | [0…1] | u |
| Arable land area (total) (e.g. of a country) | Mio. ha | A |
| Proportion of area grown with legume | [0...1] | P_L_ |
| Area grown with legumes | Mio. ha | A_L_ |
| **N rates** |  |  |
| BNF (Biological Nitrogen Fixation) | kg ha^-1^ a^-1^ N | b |
| atmospheric N deposition | kg ha^-1^ a^-1^ N | d |
| N from free-living bacteria | kg ha^-1^ a^-1^ N | f |
| N losses | kg ha^-1^ a^-1^ N | l |
| Balance rate of N produced on the area of legumes | kg ha^-1^ a^-1^ N | n_L_ |
| Balance rate of N produced on the area of non-legumes | kg ha^-1^ a^-1^ N | n_M_ |
| Balance rate of N produced on the total arable area (i.e. from legumes and non-legumes), maximally available for the area of the non-legume | kg ha^-1^ a^-1^ N | m |
| Yield saturation point (further N increase does not lead to increase in yield) | kg ha^-1^ a^-1^ N | s |
| **Yields** |  |  |
| yield of non-legume at zero N input BNF | t ha^-1^ a^-1^ | y_Mz_ |
| yield level of the non-legume, organic system | t ha^-1^ a^-1^ | y_Mo_ |
| yield level of the non-legume, conventional (FM @14% DMC) | t ha^-1^ a^-1^ | y_Mc_ |
| yield level of legume | t ha^-1^ a^-1^ | y_L_ |
| **Production** |  |  |
| Production of non-legume, organic system | t a^-1^ | Y_Mo_ |
| Production of non-legume, conventional system | t a^-1^ | Y_Mc_ |
| **OC: Ratio, i.e. ratio of organic production to conventional production of non-legume** | **[0...x]** | **R_Y_** |

The total arable area A is divided into a legume crop (proportion P_L_) and a non-legume crop (proportion P_M_ = *1* – P_L_). Nitrogen (N) input *rates* are defined as amounts of N per unit area per cropping year, here expressed in units of kg ha^-1^ a^-1^. *On* the area with legumes, **net input rates of N** from all sources including BNF, as well as losses, are

n_L_ = b + d + f – *l* (eqn. 1.1)

On the area without legumes, i.e. without BNF, net rates are

n_M_ = d + f – *l* (eqn. 1.2)

The rate of N from legumes *for* the area of the non-legume is

m = n_L_ AP_L_/(AP_M_) = n_L_ P_L_ /(1 – P_L_) (eqn. 1.3)

The yield level (in t ha^-1^ a^-1^) of the non-legume M in the legume-supported cropping system is then

y_Mo_ = y_Mz_ + m/u if m < s and y_Mo_ = y_Mz_ + s/u, else (eqn. 1.4)

with s being the saturation point, i.e. where a further increase in m does not lead to an increase in yield. The yield y_Mz_ of the non-legume at zero N input from BNF can be calculated as y_Mz_ = n_M_/u. (This value can be compared with empirically found values of non-legume yield at zero N fertilizer input). Thus,

y_Mo_ = [n_M_ + n_L_ P_L_ /(1 – P_L_)]/u if m < s and y_Mo_ = (n_M_ + s)/u, else. (eqn. 1.5)

The total production Y_Mo_ of the non-legume in the legume-supported cropping system is

Y_Mo_ = y_Mo_A(1 – P_L_) (eqn. 1.6),

whereas the conventional (non-legume supported) production Y_Mc_ of the non-legume is

Y_Mc_ = y_Mc_A (eqn. 1.7).

The OC-Ratio R_Y_ of the non-legume production is the ratio between the production in the legume-supported cropping system to the production in the conventional, i.e. not legume-supported cropping system, is

R_Y_ = Y_Mo_ / Y_Mc_ (eqn. 1.8).

Combining eqns. 1.5-1.8 results in

R_Y_ = (n_M_ + m) (1 – P_L_) / (u y_Mc_) if m < s, (eqn. 1.9a) and

R_Y_ = (n_M_ + s) (1 – P_L_) / (u y_Mc_), else (eqn. 1.9b).

Values for the five variables **n_M_, b, u, y_Mc_, and s** are derived from the literature.

With rearranging equations, it can be shown that

R_Y_ = P_L_ b / (u y_Mc_) + n_M_/(u y_Mc_) if m < s, and R_Y_ = P_L_[-(n_M_+s)/(u y_Mc_)] + (n_M_+s)/(u y_Mc_)

The maximum R_YM_ is then at the point where the two linear functions of P_L_ intersect, i.e. at

P_maxL_ b / (u y_Mc_) + n_M_/(u y_Mc_) = P_maxL_ [–(n_M_ + s)/(u y_Mc_)] + (n_M_ + s)/(u y_Mc_)

This is equivalent to

P_maxL_ = s/(b + nM + s) (eqn.1.10)

Inserting P_maxL_ yields R_YM_ = (n_M_ + s) [1 – s/(b + n_M_ + s)] / (u y_Mc_)

= (n_M_ + s) (b + n_M_)/[(b + n_M_ + s) (u y_Mc_)]

## Model 2. Organic : conventional ratios for energy output from non-legume and forage legume

Further, the legume does not only contribute nitrogen to the overall production, but also other products (e.g. energy, carbon), either directly in the case of pulses (grain legumes), or, in the case of forage legumes indirectly through the animal product (e.g. through milk).

Table S3: Variable explanations for Model 2

| **Variable explanation** | **Unit** | **Symbol** |
| --- | --- | --- |
| Yield of forage legume organic system (dry matter) | kg ha^-1^ a^-1^ | y_F_ |
| Production of forage legume, organic system | t a^-1^ | Y_F_ |
| Milk conversion of forage legume, organic system (Liter of milk per kg dry matter of forage) | L kg^-1^ | z_µ_ |
| Milk yield supported by forage legume, per unit area and year | L ha^-1^ a^-1^ | y_µ_ |
| Energy content of milk | MJ L^-1^ | x_µ_ |
| Milk energy yield per unit area and year | MJ ha^-1^ a^-1^ | e_µ_ |
| Energy production from milk | GJ a^-1^ | E_µ_ |
| Nitrogen concentration of milk | kg L^-1^ N | j_µ_ |
| Nitrogen export through milk | kg ha^-1^ a^-1^ N | n_µ_ |
| Energy content of non-legume, conventional and organic system | MJ t^-1^ | x_M_ |
| Energy yield of non-legume, conventional system | MJ ha^-1^ a^-1^ | e_Mc_ |
| Energy production of non-legume, conventional system | MJ a^-1^ | E_Mc_ |
| **Ratio of organic to conventional production of energy** | **[0...x]** | **R_E_** |

The energy content of the milk yield needs to be considered in a comparison of total productivity. To this end, we calculate the caloric energy value per unit area of the forage legume. The milk energy yield e_µ_ in MJ per ha and year is

e_µ_ = y_F_ z_µ_ x_µ_ (eqn. 2.1)

Accordingly, the milk energy production E_µ_ is

E_µ_ = e_µ_ A P_L_ (eqn. 2.2)

However, with the milk there is also an export of N from the system. Therefore, the total balance of N available for the non-legume (see part 1) is reduced by (at least) n_µ_

n_L_* = b + d +f – l – n_µ_ with n_µ_ = y_F_ z_µ_ j_µ_ (eqn. 2.3)

The energy production of the non-legume in the legume-supported system is

E_Mo_ = x_M_ y_Mo_* A (1 – P_L_), with (eqn. 2.4)

y_Mo_* = [n_M_ + n_L_* P_L_ /(1 – P_L_)]/u if m < s and y_Mo_* = (n_M_ + s)/u, else. (eqn. 2.5)

The total energy production of the legume-supported system is

E_To_ = E_µ_ + E_Mo_ (eqn. 2.6)

Whereas the total energy production in the conventional system is

E_Mc_ = y_Mc_ x_M_ A (eqn. 2.7)

In this case, the new OC-Ratio is

R_E_ = E_To_ / E_Mc_ (eqn. 2.8).

Combining eqns. 2.1-2.8 results in

R_E_ = [e_µ_ P_L_ + x_M_ (n_M_ + (n_L_ – y_F_ z_µ_ j) P_L_ /(1 – P_L_))/ u (1 – P_L_)] / [y_Mc_ x_M_ ]

if m* < s (eqn. 2.9a)

and R_E_ = [e_µ_ P_L_ + x_M_ (n_M_ + s)/u (1 –P_L_)] / [y_Mc_ x_M_] else (eqn. 2.9b).

For these calculations values for the further four variables **x_M_**, **y_F ,_ z_µ_ , j_µ_** are derived from the literature (see below).

m* > s:

R = ((e_µ_– x_M_ (n_M_ + s)/u)/[y_Mc_ x_M_ ]) **P_L_** + (x_M_ (n_M_ + s)/u)/[y_Mc_ x_M_ ]

m* < s:

R_E_ = **P_L_** (1/(y_Mc_ x_M_)) [e_µ_ + (x_M_ /u) (n_L_* – n_M_)] + n_M_ /(u y_Mc_)

## Further model assumptions and data sources

1. **Animal manure**: N input from animal manure in the conventional system does not need to be replaced by BNF. This N source is either derived from the system itself (i.e. recycled) and does therefore not need to be replaced by BNF anyway; or it is an unsustainable import and cannot be added to BNF.
2. **Grain N content**: The target N content is taken from organic grain data, which tends to be lower than from conventional grain. In the model this restriction means we will tend to overestimate organic yields, since there is more grain yield per unit N. The N content in grain is assumed to be low even if N input increases (Russell 1963).
3. **Saturation point**: The N saturation point is the N level at which no further yield increase can be affected with additional N input. An analysis of yield data taken from various studies showed that the saturation point *s* is itself linearly linked to maximally achieved yield level (*s*= 21.5 *y* + 62, df = 56, R²=0.60). Since variation in *s* is strongly dependent on *y*, it was decided to model *s* using this linear equation with the reference conventional yield *y*_Mc_ as input. To model the three k-scenarios, confidence intervals around predicted values of *s* were constructed for each yield level, corresponding to each level of k. For example, for the high yield reference level of 7.5 t ha^-1^ this procedure resulted in values of 220, 224 and 229 kg ha^-1^ a^-1^ N for *s*, corresponding to the pessimistic, realistic and optimistic scenario.
4. **BNF**: With regard to BNF, the oldest source included was from 1981. Only forage legumes were included in the modelling and the data was restricted to those legume species known to have comparatively high rates of BNF, namely white clover (*Trifolium repens*), red clover (*T. pratense*) and lucerne (*Medicago sativa*).
5. **Conventional yield**: The conventional reference level for wheat yield is based on FAO data, including 83 countries with an average wheat growing area of > 50,000 ha over the period of 2005-2014. The high reference yields in Fig. 4 a –c correspond to the 95% percentile of all yield data from these countries. Simultaneously, this value (7.5 t ha^-1^) happens to equal the 10-year national average wheat yield for Germany for the period of 2004 to 2013. The moderate reference value (Fig. 4d) corresponds to the 75% percentile. [For the most recent years (2015-2019) FAO data shows an increase of wheat yield levels for the countries with >50,000 ha of wheat in this period].
6. **Absolute yield ceiling**. In some rare cases when very high BNF values were combined with high saturation points, the model generated unrealistically high yield levels for the organic wheat. A yield ceiling was therefore introduced at 12.7 t ha^-1^ to cap organic yields in the model. This value is the 90%-percentile of *conventional* wheat yield data obtained from the region of highest yields in Germany (N = 16, official variety testing programme, 2011-2015), thereby using an extremely optimistic scenario for the absolute (non N-limited) upper limit of organic wheat yields. The value is also in line with the 12 t ha^-1^ estimated as an upper ceiling for conventional wheat yields based on current rates of genetic improvement (Hall & Richards 2013. Field Crops Res. 143, 18-33).
7. **Milk N content**: The N content in milk is partly based on Steinshamn &, Thuen 2008. Livestock Science 119: 202–215, and is kept fixed in the model.

## Modelling oat (*Avena sativa*) as an alternative crop species

As an alternative non-legume crop species, oat was used in the same modelling framework. Model input values for atmospheric N deposition, N from free-living bacteria and N losses were the same as in the wheat model. Further input values are listed in table S4. Model outputs are shown in Table S5, depending on the BNF-level and the reference yield level.

Table S4: Input data for oat model

| **Input data** | **Type of value** | **Number of values** | **Value** | **Sources** |
| --- | --- | --- | --- | --- |
| N content in grain (%)^a^ | Median | 27 | 1.66% | [1, 2] |
| N saturation point (kg ha^-1^) | Median | 10 | 96 | [3-9] |
| Reference grain yield (t ha^-1^) | 95%-Percentile | 31 | 4.64 | [11] |
|  | Median |  | 2.30 |  |

^a^: N to protein conversion factor: 6.25

Sources: [1] Dimberg LH et al. 2005. *Ambio* 34:331-337, [2] Pużyńska K. et al. 2021. *Agriculture* 11:79; [3] Zimmer J et al. 2005. *Arch Agron Soil Sci* 51: 135-149; [4]: Ma BL et al. 2012. *Can J Plant Sci* 92: 1213-1222; [5] Bukan M et al. 2015. *Poljoprivreda* 21:15-21; [6] Mantai RD et al. 2015. *Afr J Agr Res* 10:3773-3781; [7] Pecio A, Bichonski A. 2010. *Pol J Env Stud* 19: 1297-1305; [8] Givens DI et al. 2004. *Anim Feed Sci Technol* 113:169-181; [9] Welch RW, Yong YY. 1980. *J Sci Food Agr* 31:541-548; [10] FAOSTAT http://www.fao.org/faostat/

Table S5: Model output for oat model

| **BNF level** | **Model output variable^a^** | **High reference yield (95-percentile)** | **Moderate reference yield (median)** |
| --- | --- | --- | --- |
| Pessimistic: 132 kg ha^-1^ | O:C_dm_ | 0.81 | 1.64 |
|  | O:C_dm_ [P_L_≤0.33] | 0.69 | 1.40 |
| Realistic: 186 kg ha^-1^ | O:C_dm_ | 0.92 | 1.86 |
|  | O:C_dm_[P_L_≤0.33] | 0.92 | 1.86 |
| Optimistic: 282 kg ha^-1^ | O:C_dm_ | 1.03 | 2.08 |
|  | O:C_dm_[P_L_≤0.33] | 1.03 | 2.08 |

^a^: Maximal OC-Ratio with unrestricted proportion of legumes P_L_ and maximal OC-Ratio for yield with P_L_ capped at 0.33.

The OC-Ratios achieved here for oats are much higher than shown for wheat; this is mainly the consequence of a much lower reference yield level in oats than in wheat, but also, to a lesser degree because of a lower fraction of N in the grain of oats than in wheat grain. Further, however, the oats crop has a lower N saturation point than wheat, so that especially at high BNF levels, further yield gains cannot be achieved by expanding the legume area, because additional N available through a higher share of legumes in the land cannot be translated into additional yield beyond the N saturation point of oats. The generally higher OC-ratios in oats may be seen to be promising from an organic production perspective. It may therefore be considered to replace wheat with oats in general scenario of non-legume cropping. However, in this context another important criterion for the decision which non-legume crop to grow is the *absolute* yield level of the (legume-supported) cereal.

## Sensitivity to variations in grain N content, N balance on non-legume area, and saturation point

Fig. S1: Response of O:C ratio for wheat yield to variations in u (grain N content), with all other input factors held equal (y_MC_=7.5 t ha^-1^; b = 230 kg ha^-1^; s tied to y_MC_). Abbreviations see Tables S1, S2


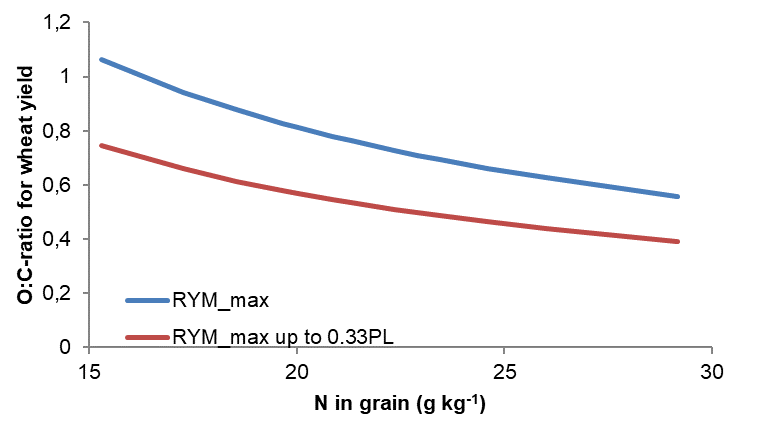


Fig. S2: Response of O:C ratio for wheat yield to variations in n_M_ (balance of N on non-legume area), with all other input factors held equal (y_MC_=7.5 t ha^-1^; u= 2,21%, b = 230 kg ha^-1^; s tied to y_MC_). Abbreviations see Tables S1, S2


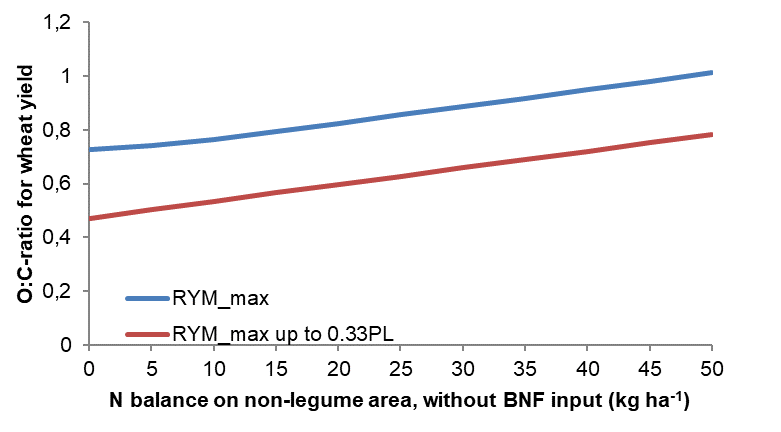


Fig. S3: Response of O:C ratio for wheat yield to variations in s (N-saturation point), with all other input factors held equal (y_MC_=7.5 t ha^-1^; u= 2,21%, n_M_ = 10 kg ha^-1^, b = 230 kg ha^-1^). Abbreviations see Tables S1, S2


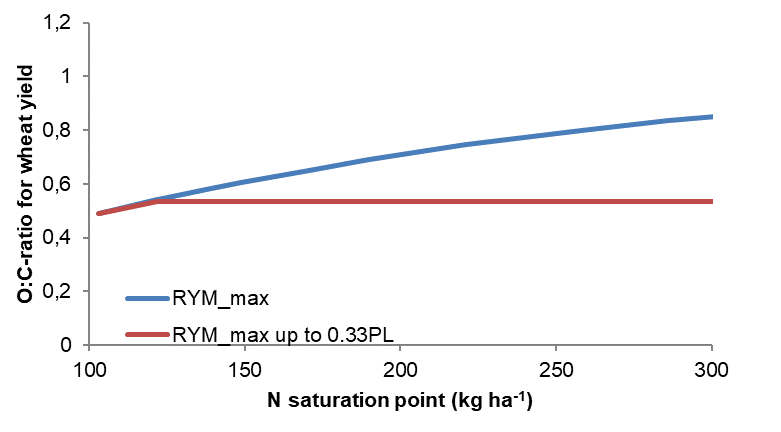

Supplement: Supplementary file 1 — Supplementary Information 1. [file 41598_2021_91940_MOESM1_ESM.docx]
